# The association between the composite dietary antioxidant index and thyroid functionality among adults in the USA: NHANES 2007–2012

**DOI:** 10.1016/j.heliyon.2024.e29082

**Published:** 2024-04-04

**Authors:** Junru Liu, Xiaofeng Lu, Jialu Song, Huijing Tong, Chaoyang Xu, Xiaotao Zhu, Xiaogang Zheng, Mingzheng Wang

**Affiliations:** a Department of Endocrinology and Metabolism, Jinhua People's Hospital, Jinhua, Zhejiang, China; b Department of Breast and Thyroid, Jinhua Central Hospital, Jinhua, Zhejiang, China; c Department of Emergency, Jinhua Central Hospital, Jinhua, Zhejiang, China; d Department of Breast and Thyroid, Jinhua Maternal and Child Health Hospital, Jinhua, Zhejiang, China

**Keywords:** Thyroid function, Composite dietary antioxidant index, NHANES

## Abstract

**Objective:**

Composite Dietary Antioxidant Index (CDAI) values serve as a summary of an individual's combined dietary antioxidant intake. Although specific antioxidants are known to reduce thyroid damage from oxidative stress, the relationship between the CDAI and thyroid function remains uncertain. The purpose of this study was thus to investigate this relationship in greater detail while focusing on a representative American adult population.

**Methods:**

A total of 6,860 subjects from the 2007–2012 NHANES cohort were included in this study. Associations between CDAI values and thyroid function were evaluated with weighted linear regression models and smoothed curve fitting. Subgroup analyses were also performed.

**Results:**

The weighted mean (SD) values for variables analyzed in this study included a CDAI of 0.13 (0.06), serum free T4 (FT4) levels of 0.80 (0.01) ng/dL, and serum total T4 (TT4) levels of 7.80 (0.03) ug/dL. Lower CDAI values were found to be associated with higher levels of FT4 and TT4 using both unadjusted and adjusted models that accounted for relevant confounders (adjusted model, FT4 β = −0.003, p = 0.005; TT4 β = −0.035, p < 0.001). This negative correlation persisted when CDAI was categorized into quartiles (FT4, p for trend = 0.014; TT4, p for trend = 0.003).

**Conclusion:**

These findings suggest that a diet rich in antioxidants, as reflected by higher CDAI scores, is associated with significant decreases in levels of free and total T4. Further analyses will be necessary to better clarify the underlying mechanisms behind these observations.

## 1 Introduction

Reactive oxygen species (ROS) are major oxidative mediators, and their excessive accumulation can lead to oxidative stress (OxS) and consequent damage to a range of molecular structures [1]. ROS are crucial regulators of thyroid activity given that they are necessary for the initiation of thyroid hormone synthesis through the oxidation of iodine [2]. Furthermore, ROS are involved in the catalytic activity of thyroid peroxidase (TPO), which is responsible for the synthesis of triiodothyronine (T3) and thyroxine (T4) within the thyroid follicles. The thyroid relies on ROS for normal function and is therefore particularly susceptible to oxidative damage due to constant exposure to these free radicals [3].

Various exogenous antioxidant molecules have been shown to help manage the excessive accumulation of ROS in organisms. Examples include curcumin [4], resveratrol [5], and oleic acid [6] derived from unsaturated fatty acids. Additionally, trace elements essential for thyroid hormone synthesis, such as iodine [7], selenium [8], and zinc [9], also act as antioxidants to mitigate ROS damage to the thyroid.

The Composite Dietary Antioxidant Index (CDAI) serves as a measure of a given individual's combined dietary antioxidant intake. Previous studies have indicated that higher CDAI levels are linked to a reduced risk of osteoporosis [10], gout [11], depression [12], hypertension [13], cancer [14], and cardiovascular death [15]. While the specific effects of particular antioxidants on the thyroid have been reported in many studies [[16], [17], [18], [19], [20], [21], [22], [23], [24], [25], [26]], the precise relationship between CDAI and thyroid function warrants further clarification. As such, this study was designed to investigate the relationship between the CDAI and thyroid function among adults in the USA based on data derived from the National Health and Nutrition Examination Survey (NHANES).

## 2 Methods

### 2.1 Study population

The NHANES is a comprehensive survey conducted in the United States that collects data regarding nationally representative samples of adults and children, including demographics, health-related behaviors, and nutrition at the individual level. The NHANES data are accessible online (www.cdc.gov/nchs/nhanes), and the analyses conducted in this study followed the principles outlined in the Declaration of Helsinki.

**Fig. 1:**
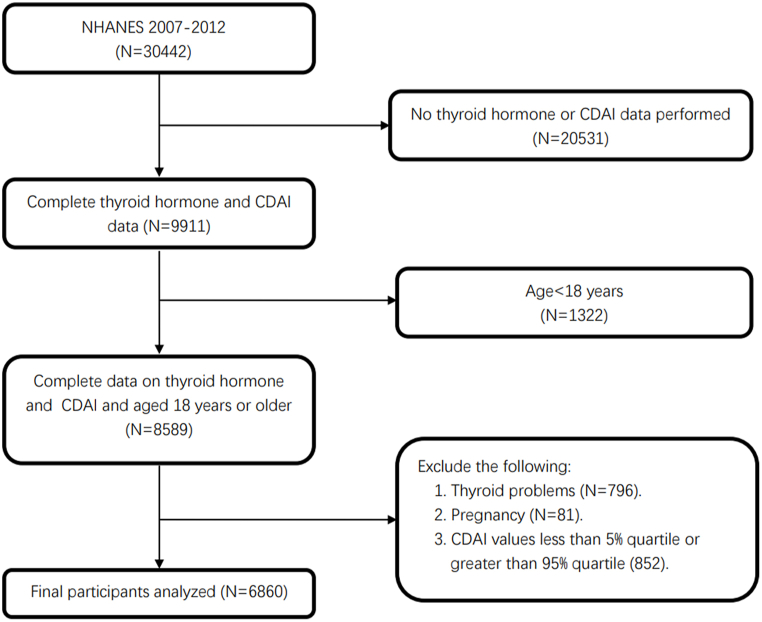
Study flowchart. NHANES, National Health and Nutrition Examination Survey.

For the present analysis, NHANES data from 2007 to 2012 were used. These data were collected with National Center for Health Statistics (NCHS) Research Ethics Review Board (ERB) approval and informed consent from all participants. This study included 6,860 subjects aged 18 years and older who had complete thyroid function and dietary data necessary for calculating CDAI values. To ensure the reliability of this analysis, we excluded participants with thyroid problems and pregnancy, and then excluded participants with CDAI scores outside the 5th and 95th percentiles. More detailed information on participant screening can be found in Fig. 1.

## 3 Measurements

### 3.1 CDAI

A 24-h dietary history interview was conducted in the Mobile Examination Center (MEC) for each participant to record dietary intake. Dietary intake was collected and calculated using the United States Department of Agriculture (USDA) dietary survey instrument, the Automated Multiple Pass Method (AMPM), which is a fully computerised recall method [[27], [28], [29], [30]]. The CDAI introduced by Wright et al. was computed based on the combined intake of several dietary antioxidants [31]. For this analysis, the CDAI incorporated six specific dietary antioxidants: Vitamin A, C, and E; manganese (Mn); selenium (Se); and zinc (Zn). The CDAI equation was as follows:

$$\text{CDAI}=\Sigma_{i=1}^{6}\frac{x_{i}-\mu_{i}}{s_{i}}$$

where $X_{i}$ represents the daily antioxidant intake, $\mu_{i}$ indicates the mean amount of these antioxidants within the study cohort, and $s_{i}$ denotes the standard deviation of $\mu_{i}$ [14, 31, 32] By leveraging these 24-hr dietary recall data, CDAI scores can provide a comprehensive evaluation of antioxidant intake at the individual level. In the context of this study, CDAI scores were treated as both continuous and categorical variables (using quartiles), thereby enabling analyses of the correlative relationships between CDAI values and thyroid function.

### 3.2 Thyroid function profile

Analyzed thyroid parameters included levels of TSH, TT3, TT4, FT3, FT4, Tg, TPOAb, and TgAb, all of which were analyzed using the Access 2 procedure with reagents purchased from Beckman Coulter. After blood samples were collected from these participants, testing was performed by a designated laboratory using samples delivered once a week. TSH was measured using third-generation two-site immunoassays (normal range: 0.34–5.6 mIU/L). FT3 levels were measured with a competitive binding immunoassay (normal range: 2.5–3.9 pg/mL), while FT4 levels were quantified with a two-step immunoassay (normal range: 0.6–1.6 ng/dL). TT4 and TT3 concentrations were determined by competitive immunoassays with respective reference ranges of 80–220 ng/dL and 5.0–12.0 μg/dL, respectively. Titers for TPOAb and TgAb were evaluated using a sequential two-step immunoassay with respective reference ranges of 0–9.0 IU/mL and 0–4.0 IU/mL. For the Access Tg assay, one-step simultaneous sandwich assays were used. For additional details on these assays, refer to the NHANES Study Team Procedural Manuals [[33], [34], [35]].

### 3.3 Covariates

Standardized questionnaires were used to gather information about age, gender, race/ethnicity, poverty-to-income ratio (PIR), alcohol consumption, smoking history, physical activity, and marital status of the study participants. Body mass index (BMI) and urinary iodine concentration (UIC) were assessed in MECs. Race/ethnicity is categorized as White, Black, Mexican, Other. Continuous variables were treated as categorical variables when performing subgroup analyses. PIR values were grouped as follows: <1 (impoverished), 1-<4 (normal), and ≥4 (affluent). Age groups were classified as ≤50 and > 50 years. BMI was used to classify individuals into three categories: <25 (normal), 25-<30 (overweight), and ≥30 kg/m^2^ (obese). UIC values were categorized as < 100 (deficient), 100-<300(normal), and ≥300 μg/L (excessive iodine intake) [36]. Marital status was classified into three groups: two (married or cohabiting), one (widowed, divorced, or separated), or never (never married). Alcohol intake in the past 12 months was classified as never, former, light, moderate, or heavy, based on the number of drinks in the past year [37]. Smoking status categories included never former, and current smokers. Physical activity level was determined based on whether participants engaged in at least 10 consecutive minutes of work, walking, biking, or recreational activity in an average week. Laboratory parameters were measured by trained technicians using standard protocols.

### 3.4 Statistical analysis

Weighted data comparisons and analyses were performed with reference to the NHANES 2007–2012 Analytical Guidelines. Multiple sample weights are provided in the available NHANES data files, including both examination and interview weights (wtmec2yr and wtint2yr, respectively), with the most appropriate weight being determined by the particular variable or variables of interest. Given that MEC samples represent a subset of the overall interview sample, the examination sample weights are used in this analysis. Thyroid analyses from the NHANES 2009–2012 cycles were a subset of individuals that underwent MEC analyses such that data from these survey cycles were analyzed with the WTSA2YR subset weighting. Appropriate utilization of these weighting values ensures that survey results can be extended to the civilian, non-institutionalized U.S. population [38].

The relationship between the CDAI and thyroid function was evaluated using unadjusted (Model 1) and adjusted (Model 2) linear regression models. Model 2 was adjusted for UIC, PIR, physical activity, age, gender, ethnicity, smoking status, alcohol intake, BMI, and marital status. Smooth curve fitting for generalized additive model regression analysis was also conducted, and subgroup analyses were utilized to assess the independent impact of CDAI on thyroid functionality. Results are given as means and standard deviations (SDs) or percentages. β-values and 95% confidence intervals (CIs) were used to report effect values. P < 0.05 was selected as significance threshold. R v4.2.2 was used to conduct all analyses with the 'nhanesR' (v0.9.4.7) and the 'survey' (v 4.1–1) packages.

## 4 Results

### 4.1 Participant characteristics

These analyses incorporated 6,860 subjects from the NHANES 2007–2012 dataset. The characteristics of individuals in each CDAI quartile are compiled in Supplementary Table 1. These subjects included 3,679 (weighted proportion: 52.62%) males and 3,181 (weighted proportion: 47.38%) females, with a weighted mean (SD) age of 44.98 (0.51) years. CDAI scores ranged from −4.51 to 7.71, with a weighted mean of 0.13 (0.06), and the ranges for each of the four CDAI quartiles were as follows: −4.51 to −2.01, −2.02 to −0.28, −0.29 to 1.94, and 1.95 to 7.71, respectively. As shown in Table 1, the levels of FT4 and TT4 differed significantly between CDAI quartiles.

**Table 1:** Thyroid function of the NHANES (2007–2012) study population in CDAI quartiles.

Variable	CDAI					*P*-value
	total	Q1	Q2	Q3	Q4	
*N*	6860	1976	1691	1662	1531	
CDAI	−4.51 to 7.71	−4.51 to −2.01	−2.02 to −0.28	−0.29 to 1.94	1.95 to 7.71	
CDAI	0.13(0.06)^a^	−3.09(0.03)	−1.16(0.01)	0.76(0.02)	3.99(0.06)	<0.001
TSH (mIU/L)	1.86(0.03)	1.85(0.05)	1.95(0.07)	1.78(0.04)	1.86(0.05)	0.2
FT3 (pg/mL)	3.20(0.01)	3.20(0.01)	3.21(0.02)	3.18(0.02)	3.22(0.02)	0.21
FT4 (ng/dL)	0.80(0.01)	0.81(0.01)	0.80(0.01)	0.78(0.01)	0.79(0.01)	<0.001
TT3 (ng/dL)	114.97(0.63)	116.13(0.99)	114.02(0.99)	113.51(0.77)	116.20(0.96)	0.05
TT4 (ug/dL)	7.80(0.03)	8.03(0.05)	7.79(0.06)	7.66(0.07)	7.70(0.06)	<0.001
TPOAb (IU/mL)	16.91(1.76)	14.09(2.13)	23.71(5.37)	16.05(3.09)	13.83(2.13)	0.26
TgAb (IU/mL)	5.60(0.83)	5.13(1.21)	6.81(1.40)	6.25(2.10)	4.20(1.10)	0.56
Tg (ng/mL)	15.23(0.54)	15.38(0.71)	16.40(1.25)	15.05(1.40)	14.09(0.56)	0.12

Abbreviations: CDAI, composite dietary antioxidant index; FT3, free triiodothyronine; FT4, free thyroxine; TSH, thyroid stimulating hormone; TT3, total T3; TT4, total T4; Tg, thyroglobulin; TPOAb, thyroid peroxidase antibody; SD, standard deviation.

a Continuous variable: weighted mean (SD).

### 4.2 Associations between thyroid function and CDAI

Table 2 demonstrates that CDAI was negatively associated with FT4 and TT4 in the unadjusted model. Using Model 2 (adjusted for UIC, PIR, physical activity, age, gender, ethnicity, smoking status, alcohol intake, BMI, and marital status), FT4 and TT4 remained negatively associated with CDAI (FT4: β = −0.003, 95% CI: −0.004 to −0.001, P = 0.005; TT4: β = −0.035.95% CI: −0.053 to −0.018, P < 0.001). This negative correlation persisted when quartiles were used to analyze CDAI as a categorical variable (FT4, P for trend = 0.014; TT4, P for trend = 0.003).

**Table 2:** The relationship between CDAI and thyroid function.

	Model 1^a^ β(95% CI)	*p*-value	Model 2^b^ β(95% CI)	*p*-value
TSH (mIU/L)				
Total CDAI	−0.006(-0.025, 0.012)	0.503	−0.011(-0.028,0.006)	0.211
Categories				
Q1	Reference		Reference	
Q2	0.098(-0.066,0.261)	0.234	0.041(-0.156,0.238)	0.551
Q3	−0.065(-0.169,0.040)	0.217	−0.092(-0.207,0.024)	0.675
Q4	0.008(-0.125,0.140)	0.907	−0.014(-0.169,0.142)	0.114
p for trend		0.511		0.211
FT3(pg/mL)				
Total CDAI	0.002(-0.003, 0.007)	0.411	0.001(-0.004, 0.006)	0.709
Categories				
Q1	Reference		Reference	
Q2	0.002(-0.044,0.048)	0.924	0.026(-0.026,0.077)	0.313
Q3	−0.024(-0.062,0.014)	0.205	−0.001(-0.038,0.037)	0.978
Q4	0.014(-0.021,0.049)	0.410	0.014(-0.025,0.053)	0.462
p for trend		0.181		0.887
**FT4(ng/dL)**				
Total CDAI	−0.003(-0.005, −0.001)	<0.001	−0.003(-0.004, −0.001)	0.005
Categories				
Q1	Reference		Reference	
Q2	−0.002(-0.015,0.011)	0.757	0(-0.013,0.013)	0.966
Q3	−0.025(-0.037,-0.014)	<0.001	−0.021(-0.035,-0.008)	0.003
Q4	−0.014(-0.029,-0.000)	0.049	−0.012(-0.026,0.003)	0.105
p for trend		0.001		0.014
**TT3(ng/dL)**				
Total CDAI	−0.018(-0.325, 0.288)	0.905	0.071(-0.223, 0.364)	0.626
Categories				
Q1	Reference		Reference	
Q2	−2.112(-4.465,0.242)	0.078	−0.630(-3.033,1.772)	0.596
Q3	−2.621(-5.045,-0.196)	0.035	−1.103(-3.246,1.040)	0.301
Q4	0.075(-2.419,2.568)	0.952	1.030(-1.400,3.459)	0.393
p for trend		0.012		0.119
**TT4(ug/dL)**				
Total CDAI	−0.047(-0.064, −0.030)	<0.001	−0.035(-0.053, −0.018)	<0.001
Categories				
Q1	Reference		Reference	
Q2	−0.242(-0.383,-0.102)	0.001	−0.224(-0.390,-0.057)	0.010
Q3	−0.369(-0.547,-0.191)	<0.001	−0.325(-0.516,-0.135)	0.002
Q4	−0.334(-0.476,-0.191)	<0.001	−0.248(-0.397,-0.098)	0.002
p for trend		<0.001		0.003

Abbreviations: CDAI, composite dietary antioxidant index; TSH, thyroid-stimulating hormone; FT3, free triiodothyronine; FT4, free thyroxine; TT3, total T3; TT4, total T4.

a Model 1: no covariates were adjusted.

b Model 2: age, gender, race/ethnicity, poverty-to-income ratio, marital status, body mass index, alcohol use, smoking, urinary iodine concentration, and physical activity were adjusted.

**Fig. 2:**
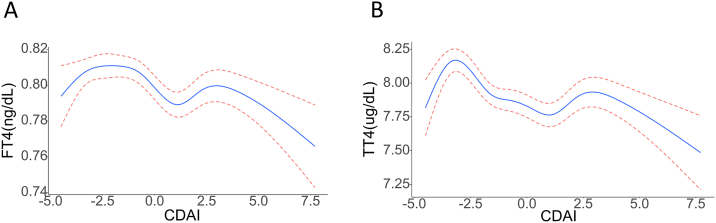
Association between thyroid function and CDAI in a generalized additive model (GAM) and a segmented linear model. A, smoothed curve of FT4 with CDAI; B, smoothed curve of TT4 with CDAI. The blue solid line represents the smoothed curve fit between the variables, while the 95% CI of this fit is indicated by the pink dashed line. (For interpretation of the references to colour in this figure legend, the reader is referred to the Web version of this article.)

Further generalized additive model analyses indicated that FT4 and TT4 levels were also negatively correlated with CDAI, and the associations between FT4, TT4, and CDAI conformed to oblique M-shaped curves (Fig. 2A and B). The blue and pink lines respectively correspond to the smooth curve fitting and 95% CI for the relationships between these variables.

### 4.3 Subgroup analyses

**Fig. 3:**
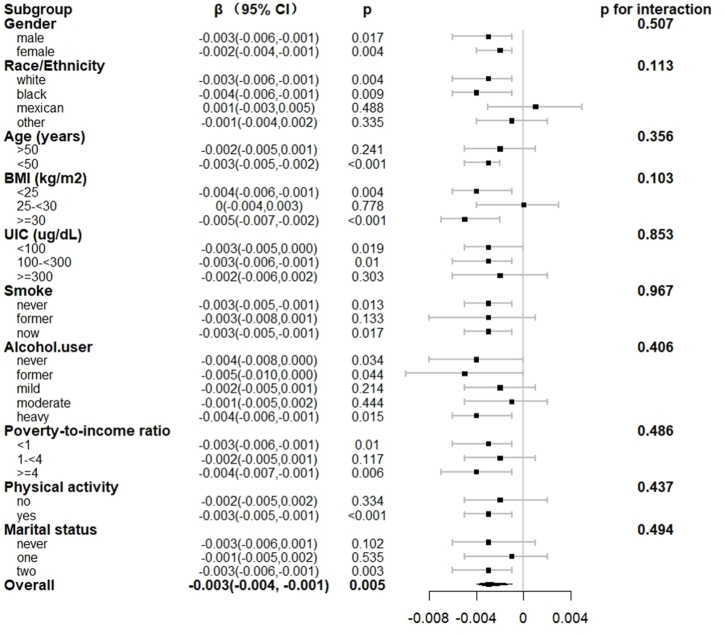
Relationship between FT4 and CDAI in each subgroup. Each subgroup adjusted for all factors (gender, age, race/ethnicity, marital status, BMI, poverty income ratio, smoking status, alcohol consumption status, UIC, physical activity) except the subgroup factor itself.

**Fig. 4:**
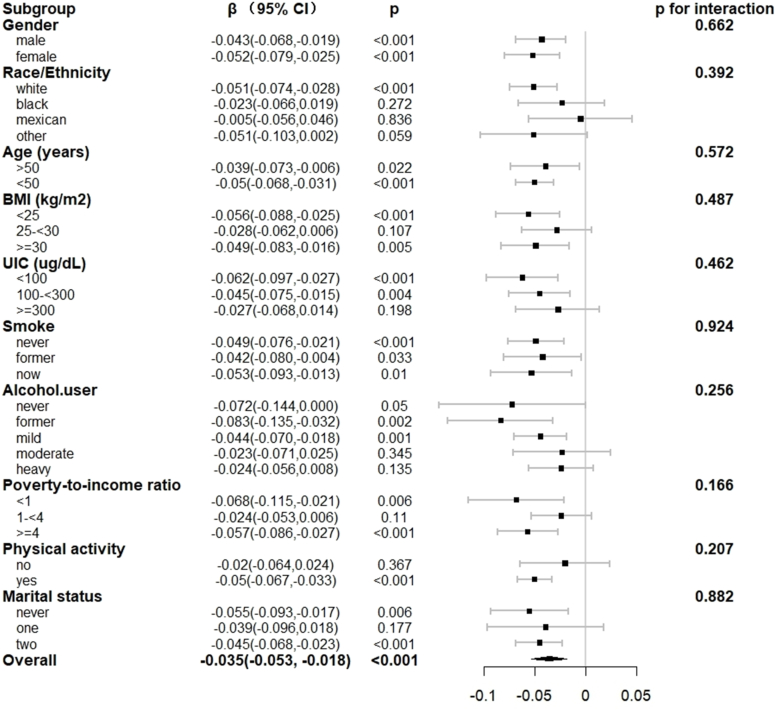
Relationship between TT4 and CDAI in each subgroup. Each subgroup adjusted for all factors (gender, age, race/ethnicity, marital status, BMI, poverty income ratio, smoking status, alcohol consumption status, UIC, physical activity) except the subgroup factor itself.

We also performed subgroup analyses by grouping continuous variables into categorical variables, and then performing subgroup analyses of FT4, TT4, and CDAI levels for each categorical variable. As shown in Fig. 3, Fig. 4, although the results of these analyses indicated a negative correlation in most subgroups, further testing for interactions revealed no statistically significant differences between subgroups (P for interaction >0.05).

## 5 Discussion

This study was developed to assess the relationship between CDAI and thyroid function through analyses of the NHANES dataset, which includes a cohort of representative adults from the USA. Our study excluded pregnant individuals and those with thyroid problems. We selected eight indicators of thyroid function: TSH, FT3, TT3 FT4, TT4, TG, TPOAb, and TGAb. Univariate analyses indicated that only FT4 and TT4 levels were negatively related to CDAI values. These negative relationships remained significant even when adjusting for relevant confounders. Overall, dietary antioxidant intake was thus associated with lower FT4 and TT4 levels.

This is the first investigation to our knowledge of the correlation between CDAI levels and thyroid function. Previous studies have focused on the individual effects of dietary antioxidants on thyroid function, including vitamins A, C, and E, as well as trace minerals like zinc, and selenium. However, none of these studies have employed a comprehensive antioxidant index like the CDAI to assess the relationship with thyroid function. In a typical daily diet, it is often unrealistic to achieve sufficient intake of a single antioxidant, and it is more likely that an assortment of foods containing various antioxidants will instead be consumed. Additionally, foods like tomatoes, which are rich in antioxidants such as vitamins A, E, C, and manganese, do not contain a single antioxidant component [39]. Therefore, a comprehensive investigation of the effects of dietary antioxidants on thyroid function is warranted. Our results suggest that a diet richer in antioxidants, as indicated by higher CDAI levels, is associated with lower levels of FT4 and TT4.

There is a significant body of research in this field that explores the associations, causality, and mechanisms underlying how these individual nutrients affect thyroid function. Vitamin A appears to have a particularly important role in regulating thyroid homeostasis, either independently or in conjunction with other micronutrients, especially iodine [16]. Rabbani et al. discovered that administration of vitamin A increased FT4 levels in patients with hypothyroidism [17]. Zimmermann et al. also found that vitamin A supplementation, along with iodized salt, significantly reduced thyroid volume and serum TSH levels [18]. Vitamin C, known for its antioxidant properties, is involved in maintaining various bodily functions [19]. In a study conducted by Seven et al. vitamin C supplementation was shown to alleviate oxidative stress in hyperthyroid patients treated with propylthiouracil [20]. Vitamin E is primarily known for its role in protecting polyunsaturated fatty acids (PUFAs), cell membranes, and low-density lipoproteins (LDL) from free radical oxidation [21]. Additionally, several micronutrients influence thyroid function. Selenium is a key element involved in the biosynthetic production and metabolic processing of thyroid hormones [22]. Selenium deficiency reduces the conversion of T4 to T3 [23]. Lower serum selenium levels have been linked to newly diagnosed Graves’ disease and autoimmune hypothyroidism [24]. Both hypothyroidism and hyperthyroidism have been tied to low zinc levels [25]. Another study revealed a strong association between serum manganese levels and thyroid hormones, as high manganese concentrations led to a reduction in the concentrations of free T3 and FT4, thereby contributing to the incidence of hypothyroidism [26]. These findings on the relationship between intake of individual antioxidants and thyroid function are inconsistent, which may be related to the fact that different antioxidants do not have the same mechanism of action on the thyroid or thyroid hormones. The CDAI is a composite measure of intake of six antioxidants, and its relationship with the thyroid may be more complex. Our analysis using general additive modeling showed a relationship between CDAI and FT4 and TT4 "M" curves. More and more in-depth studies are needed to elucidate the mechanism of this relationship.

Several factors including genetics [40], age, gender [41], stress [42], medication use [43], and diet all impact thyroid function. To investigate potential differences between subgroups, we conducted subgroup analyses and tested for interactions. However, the p-values for the interactions of FT4, TT4, and CDAI between the subgroups were all >0.05, consistent with a lack of any significant differences between these subgroups with respect to the relationships among these variables.

This study is subject to multiple limitations. For one, these analyses were cross-sectional in design such that additional longitudinal research will be required to probe any potential causal relationships between CDAI values and thyroid function. Moreover, all measures of thyroid function were based on data collected at a single time point, potentially shaping the apparent levels of thyroid function [44], and the failure to specify a consistent time for sample collection may restrict any reliable effort to interpret these results. CDAI scores are also calculated from a single 24-h dietary recall, which may be subject to recall bias and seasonal variation, and the 24-h dietary data collected on this occasion may not correspond to the usual dietary profile. Finally, these results may have been influenced by additional confounding variables not evaluated herein. Despite these limitations, these results nonetheless offer strong population-level insight into the relationship between thyroid function and CDAI values among American adults.

## 6 Conclusion

Our study examined the relationship between the CDAI and thyroid function in a nationally representative population of US adults. We found that higher CDAI scores, indicative of an antioxidant-rich diet, were related to reduced levels of FT4 and TT4. Further research will be required to clarify the underlying mechanisms and to explore the clinical implications of these findings.

## Ethics declarations

This study was reviewed and approved by NCHS Research Ethics Review Board (ERB). All participants (or their proxies/legal guardians) provided informed consent to participate in the study.

## Data availability statement

Publicly available datasets were analyzed in this study. This data can be found here: www.cdc.gov/nchs/nhanes/.

## Funding

This project was supported by the Public Interest Jinhua Science and Technology Research Program [2021-04-008 and 2021-04-219].

## CRediT authorship contribution statement

**Junru Liu:** Writing – review & editing, Writing – original draft, Project administration, Funding acquisition, Conceptualization. **Xiaofeng Lu:** Visualization, Supervision, Formal analysis, Data curation. **Jialu Song:** Visualization, Methodology, Investigation. **Huijing Tong:** Resources, Formal analysis. **Chaoyang Xu:** Validation, Methodology. **Xiaotao Zhu:** Software, Methodology. **Xiaogang Zheng:** Validation, Software, Resources. **Mingzheng Wang:** Writing – review & editing, Writing – original draft, Validation, Software, Project administration, Funding acquisition, Data curation.

## Declaration of competing interest

The authors declare that they have no known competing financial interests or personal relationships that could have appeared to influence the work reported in this paper.

## Abbreviations

NHANES: National Health and Nutrition Examination Survey
CDAI: composite dietary antioxidant index
FT3: free triiodothyronine
FT4: free thyroxine
TSH: thyroid stimulating hormone
TT3: total triiodothyronine
TT4: total thyroxine
Tg: thyroglobulin
TPOAb: thyroid peroxidase antibody
SD: standard deviation
ROS: reactive oxygen species
NCHS: National Center for Health Statistics
BMI: body mass index
UIC: urinary iodine concentration
GAM: generalized additive model
PUFAs: polyunsaturated fatty acids
LDL: low-density lipoproteins
