# Supplementary material for: The association between the composite dietary antioxidant index and thyroid functionality among adults in the USA: NHANES 2007–2012
Source: Heliyon. 2024 Apr 4;10(7):e29082. doi: 10.1016/j.heliyon.2024.e29082 (PMC11015128; doi:10.1016/j.heliyon.2024.e29082)
Supplement: Multimedia component 1 [file mmc1.docx]

**Supplementary table 1 Characteristics of the NHANES (2007-2012) study population in CDAI quartiles.**

| **Characteristics** | **CDAI** | | | | | ***P*-value** | |
| --- | --- | --- | --- | --- | --- | --- | --- |
|  | **total** | **Q1** | **Q2** | **Q3** | **Q4** |  |  |
| *N* | 6860 | 1976 | 1691 | 1662 | 1531 |  | |
| CDAI | -4.51 to 7.71 | -4.51 to -2.01 | -2.02 to -0.28 | -0.29 to 1.94 | 1.95 to 7.71 |  |  |
| Gender |  |  |  |  |  | 0.69 | |
| Male | 3679(52.62)^a^ | 1057(51.76) | 941(54.20) | 883(51.91) | 798(52.62) |  | |
| Female | 3181(47.38) | 919(48.24) | 750(45.80) | 779(48.09) | 733(47.38) |  | |
| Race/Ethnicity |  |  |  |  |  | 0.01 | |
| White | 3071(67.84) | 853(63.71) | 751(69.02) | 751(67.87) | 716(70.75) |  | |
| Black | 1409(11.17) | 433(13.34) | 336(10.12) | 331(10.76) | 309(10.44) |  | |
| Mexican | 1152( 8.62) | 319(7.95) | 307(9.04) | 290(9.63) | 236(7.85) |  | |
| Other | 1228(12.38) | 371(15.01) | 297(11.83) | 290(11.74) | 270(10.96) |  | |
| Age (years) | 44.98(0.51)^b^ | 45.45(0.58) | 46.45(0.58) | 45.11(0.67) | 42.91(0.86) | < 0.001 | |
| Age (years) |  |  |  |  |  | 0.01 | |
| <50 | 3863(62.83) | 1007(60.76) | 922(59.36) | 977(63.30) | 957(67.87) |  | |
| >50 | 2997(37.17) | 969(39.24) | 769(40.64) | 685(36.70) | 574(32.13) |  | |
| BMI (kg/m^2^) | 28.45(0.15) | 28.47(0.23) | 28.62(0.23) | 28.52(0.38) | 28.17(0.29) | 0.59 | |
| BMI (kg/m^2^) |  |  |  |  |  | 0.33 | |
| <25 | 2076(32.73) | 589(34.31) | 468(30.88) | 486(31.83) | 533(35.19) |  | |
| 25-29.9 | 2313(33.15) | 657(30.93) | 589(33.14) | 605(35.82) | 462(33.95) |  | |
| >=30 | 2391(33.16) | 699(34.76) | 618(35.98) | 558(32.35) | 516(30.86) |  | |
| UIC (ug/dL) | 231.39(11.02) | 286.85(36.00) | 217.30(12.14) | 216.22(12.30) | 205.21(13.45) | 0.22 | |
| UIC (ug/dL) |  |  |  |  |  | 0.57 | |
| <100 | 2143(34.04) | 602(35.37) | 532(33.90) | 527(33.49) | 482(36.10) |  | |
| 100-299 | 3276(47.26) | 956(47.77) | 792(48.20) | 796(47.65) | 732(49.13) |  | |
| >300 | 1245(16.77) | 362(16.86) | 321(17.89) | 302(18.86) | 260(14.77) |  | |
| Smoke |  |  |  |  |  | 0.002 | |
| never | 3489(52.68) | 924(48.73) | 839(52.66) | 906(58.01) | 820(59.70) |  | |
| former | 1621(23.32) | 494(24.11) | 416(26.01) | 382(23.65) | 329(23.23) |  | |
| now | 1387(20.16) | 455(27.16) | 350(21.33) | 303(18.34) | 279(17.07) |  | |
| Alcohol. User |  |  |  |  |  | < 0.001 | |
| never | 825( 8.99) | 276(12.28) | 200( 9.53) | 191( 9.39) | 158( 7.99) |  | |
| former | 1159(13.67) | 393(17.05) | 285(14.93) | 268(14.87) | 213(12.78) |  | |
| mild | 1945(31.05) | 511(29.58) | 514(38.27) | 459(31.41) | 461(36.18) |  | |
| moderate | 918(16.40) | 235(16.25) | 197(14.01) | 253(20.02) | 233(21.32) |  | |
| heavy | 1343(21.58) | 376(24.84) | 339(23.26) | 340(24.30) | 288(21.73) |  | |
| Marital status |  |  |  |  |  | 0.06 | |
| never married | 1165(18.44) | 356(21.95) | 291(19.22) | 272(17.64) | 246(17.92) |  | |
| widowed, divorced, or separated | 1384(16.01) | 447(19.65) | 315(14.53) | 338(17.04) | 284(15.39) |  | |
| married or cohabiting | 3949(61.71) | 1070(58.41) | 998(66.25) | 981(65.31) | 900(66.68) |  | |
| Physical activity |  |  |  |  |  | < 0.001 | |
| no | 1763(20.38) | 600(25.93) | 468(21.61) | 365(17.12) | 330(16.88) |  | |
| yes | 5097(79.62) | 1376(74.07) | 1223(78.39) | 1297(82.88) | 1201(83.12) |  | |
| Poverty-to-income ratio | 2.96(0.06) | 2.64(0.08) | 3.06(0.08) | 3.11(0.07) | 3.04(0.07) | < 0.001 | |
| Poverty-to-income ratio |  |  |  |  |  | < 0.001 | |
| <1 | 1355(14.28) | 445(20.08) | 314(13.68) | 317(13.34) | 279(14.47) |  | |
| 1-4 | 3331(45.54) | 1007(51.75) | 836(50.45) | 788(48.30) | 700(45.73) |  | |
| >=4 | 1581(33.04) | 348(28.17) | 389(35.86) | 420(38.36) | 424(39.79) |  | |

^a^ Categorical variable: actual frequencies (weighted percentages).

^b^ Continuous variable: weighted mean (SD).

Abbreviations: CDAI, composite dietary antioxidant index; FT3, free triiodothyronine; FT4, free thyroxine; TSH, thyroid stimulating hormone; TT3, total T3; TT4, total T4; Tg, thyroglobulin; TPOAb, thyroid peroxidase antibody; BMI, body mass index; UIC, urinary iodine concentration; SD, standard deviation.
